# Comparison of upper airway patency in patients with mild obstructive sleep apnea during dexmedetomidine or propofol sedation: a prospective, randomized, controlled trial

**DOI:** 10.1186/s12871-018-0586-5

**Published:** 2018-09-05

**Authors:** Hyun-Jung Shin, Eun-Young Kim, Jung-Won Hwang, Sang-Hwan Do, Hyo-Seok Na

**Affiliations:** 1 Department of Anesthesiology and Pain Medicine, Seoul National University Bundang Hospital, 82, Gumi 173, Bundang, Seongnam, Gyeonggi 13620 South Korea; 2 Department of Anesthesiology and Pain Medicine, Daerim St. Mary’s Hospital, Seoul, South Korea

**Keywords:** Dexmedetomidine, Obstructive sleep apnea, Propofol, Sedation

## Abstract

**Background:**

In addition to propofol, dexmedetomidine is a suitable alternative for intraoperative sedation in procedures requiring regional anesthesia. To date, however, little is known about the influences of each drug on upper airway patency. Accordingly, the authors investigated differences between dexmedetomidine and propofol sedation in the occurrence of upper airway obstruction and requirements for airway intervention in patients with mild obstructive sleep apnea.

**Methods:**

Patients with an apnea/hypopnea index of 5–14/h according to Watch-PAT 200 analysis were enrolled in this study. Spinal anesthesia was routinely performed for surgery. Intraoperative sedation was initiated using either dexmedetomidine or propofol infusion at a level of modified observer’s assessment of alertness/sedation scale 3. The primary outcome was the proportion of patients exhibiting signs of upper airway obstruction. A sign of upper airway obstruction was defined as no detection of end-tidal carbon dioxide for at least 10 s despite respiratory efforts.

**Results:**

A total of 50 patients were included in the final analysis (dexmedetomidine [*n* = 26]; propofol [*n* = 24]). During the intraoperative sedation period, there was a significantly lower proportion of patients exhibiting signs of upper airway obstruction in the dexmedetomidine group than in the propofol group (11.5% vs. 41.7%, *P* = 0.035). An artificial airway was inserted in 1 patients (3.8%) and 5 patient (20.8%) in the dexmedetomidine and propofol groups, respectively (*P* = 0.093).

**Conclusion:**

Dexmedetomidine sedation was associated with a lower incidence of upper airway obstruction than propofol sedation in patients with mild obstructive sleep apnea.

**Trial registration number:**

Clinical trials.gov (NCT02993718): Retrospectively registered.

## Background

Various agents have been used to sedate patients in operating rooms, intensive care units, procedure rooms, and diagnostic rooms. When sedation is induced, close attention is required for potential adverse events such as upper airway obstruction, hypoventilation, or apnea, as well as for any cardiovascular complications [1, 2]. Obstructive sleep apnea (OSA) is a challenging condition during intraoperative sedation. OSA patients have been associated with higher rates of postoperative complications, including significant postoperative hypoxemia [3, 4].

Dexmedetomidine acts as a selective α_2_ adrenergic receptor agonist, with both sedative and analgesic effects [5]; thus, it has been favored in procedural sedation or monitored anesthesia care [6–8]. Moreover, dexmedetomidine has been known to induce sleep characteristics similar to physiological sleep [9, 10], and to be less associated with sleep apnea/hypopnea and respiratory depression [10]. In contrast, despite the advantages of propofol, such as rapid onset, short duration, and clear recovery, it has been known to cause respiratory depression, which can be exacerbated when coadministered with opioid [11, 12].

In children with OSA, dexmedetomidine sedation has less effect on airway patency and protective reflexes [13]. In adults, however, less evidence is available. To date, there has been no direct investigative comparison of which drug is better suited in OSA patients for intraoperative sedation during regional anesthesia.

We hypothesized that dexmedetomidine sedation could achieve a more patent airway; accordingly, we evaluated the occurrence of upper airway obstruction or the requirement for airway intervention in patients with mild OSA during intraoperative dexmedetomidine or propofol sedation.

## Methods

Ethics approval for this study (B-1411/275–003) was granted by the institutional review board (Seoul National University Bundang Hospital, Seoul, South Korea) on January 4, 2015. This protocol was registered at ClinicalTrial.gov (NCT02993718) on December 2016. Adult patients, who were scheduled to undergo elective surgery under spinal anesthesia, were initially screened for inclusion in this study. The exclusion criteria were as follows: American Society of Anesthesiologists physical status III-V; body mass index ≥35 kg/m^2^; refusal of intraoperative sedation; lateral decubitus or prone positioning during surgery; congenital or acquired anatomical problem of upper respiratory tract; drug intoxication; chronic alcoholics, or psychotic disorder. Written informed consent was obtained from all patients.

Patients wore the Watch-PAT 200 (Itamar Medical Ltd., Caesarea, Israel) on their wrist and index finger the night before surgery. The Watch-PAT 200, is a wrist-worn device equipped with a non-invasive finger-mounted probe, and is as useful as polysomnography for analyzing sleep patterns and diagnosing OSA [14]. Patients with an apnea/hypopnea index of 5–14 events/h according to the Watch-PAT 200 recording were finally enrolled in this study, and were assigned to either the dexmedetomidine group or the propofol group by one anesthesiologist who did not attend anesthetic care. Randomization was performed by computer-generated group allocation.

On arrival to the operating room, vital signs, including non-invasive arterial pressure, electrocardiogram, and pulse oximetry, were monitored. Spinal anesthesia was performed in a routine manner. After confirming the appropriate sensory and motor block, sedative drugs were administered to the patients. Each patient was positioned supine with a pillow 3 cm high. In the dexmedetomidine group, 0.5 μg/kg of dexmedetomidine was administered over a 10-min period, which was followed by continuous infusion in the range of 0.2–0.8 μg/kg/h. In the propofol group, propofol was infused via a target-controlled infusion device within the range 0.5–2.0 μg/ml. The sedation level was maintained according to the modified observer’s assessment of alertness/sedation (OAA/S) scale 3 (response to calling loudly or repeatedly) in all patients [15], which was assessed every 10 min. During the sedation period, oxygen was supplied to patients via face mask and, concurrently, end-tidal carbon dioxide (ETCO_2_) was monitored using the IntelliVue MP60 monitor (Philips, Boeblingen, Germany).

Signs of upper airway obstruction were defined as no detection of ETCO_2_ despite inspiratory chest wall movement. If signs of upper airway obstruction were detected, the 3 cm pillow was replaced with a 6 cm pillow to elevate the patient’s head and extended the patient’s head using a cervical roll to improve airflow through the upper airway. If the signs of upper airway obstruction persisted despite this maneuver, an artificial airway was inserted. An independent investigator monitored events of airway obstruction.

Propofol was discontinued at the end of surgery; however, dexmedetomidine was discontinued earlier, approximately 30 min before the end of surgery. After cessation of each drug, the modified OAA/S scale was evaluated every 5 min until the scale reached 5. Patients were transferred to the post-anesthetic care unit (PACU). A modified Aldrete’s scoring system was applied for discharge from the PACU. Most patients could not move their lower extremities voluntarily due to spinal anesthesia; thus, 9 of 10 points were the criteria for discharge from the PACU. In addition, when the modified OAA/S scale reached to 5, and when there was no acute operation-related complication related with operation, spinal anesthesia, and sedation, patients could be discharged from the PACU.

The primary outcome was the proportion of patients who exhibited signs of upper airway obstruction during the intraoperative sedation period. The secondary outcomes included recovery time from discontinuing each sedative drug to reach the modified OAA/S scale 5, the duration of PACU stay, and hemodynamic changes during sedation and the recovery period.

To calculate sample size, it was assumed that the incidences of upper airway obstruction requiring any airway manipulation were approximately 16% in the dexmedetomidine group and 54% in the propofol group, which were representative of pilot data from our center. Assuming an attrition rate of 10%, a total of 52 patients were determined to be an adequate sample size to aim for 80% power and 5% type-1 error.

Data are presented as mean ± SD or number (proportion) as appropriate. The Shapiro-Wilk test was performed to verify the normality of the data. Continuous variables were analyzed using the Student’s *t*-test or Mann-Whitney U test, and categorical data were analyzed using the χ^2^ test or Fisher’s exact test, as appropriate. Changes in mean arterial pressure and heart rate were analyzed using repeated-measures ANOVA. SPSS version 21.0 (IBM Corporation, Armonk, NY, USA) was used for statistical analyses; differences with *P* < 0.05 were considered to be statistically significant.

## Results

**Fig. 1:**
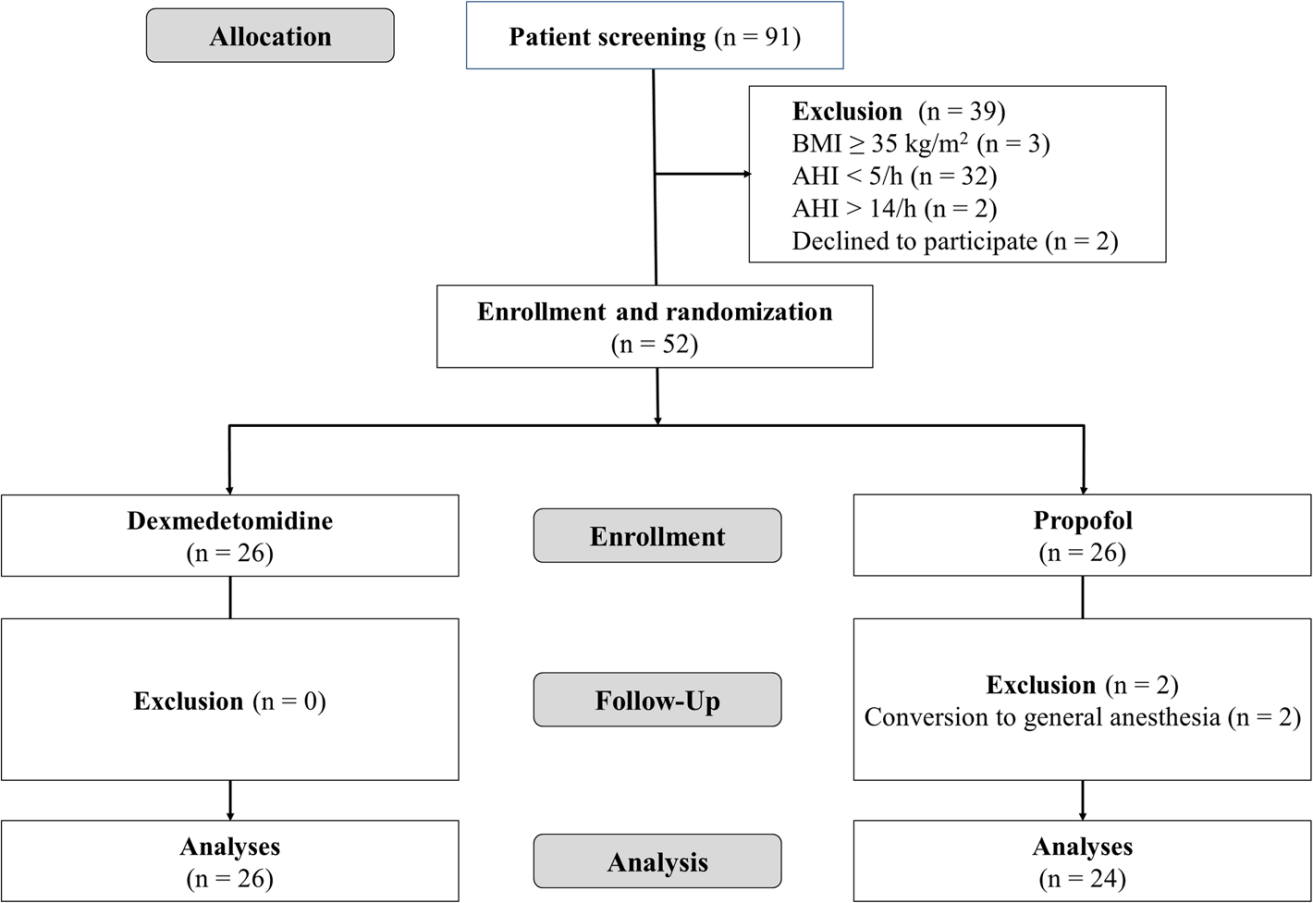
Flow chart of patients’ enrollment. AHI, apnea/hypopnea index; BMI, body mass index

From January 2015 to June 2017, a total of 52 patients were enrolled in the present study. Two patients dropped out due to conversion of the anesthesia method from spinal to general (Fig. 1). Characteristics of the patients and preoperative Watch-PAT 200 data were summarized in Table 1. The profiles of operation, anesthesia, and recovery, and intraoperative vasopressor requirement were comparable between the two groups (Table 2).

**Table 1:** The characteristics of patients and preoperative WatchPAT200 analyses

	Dexmedetomidine (*n* = 26)	Propofol (*n* = 24)
Age (years)	56.2 ± 10.0	53.6 ± 11.4
Gender (Male/female)	18/8 (69.2/30.8)	15/9 (62.5/37.5)
Height (cm)	164.3 ± 8.9	163.3 ± 9.6
Weight (kg)	72.2 ± 11.7	68.9 ± 10.4
BMI (kg/m^2^)	26.7 ± 3.7	25.8 ± 3.3
ASA (I/II)	9/17 (34.6/65.4)	12/12 (50.0/50.5)
WatchPAT200 analyses		
Mean SpO_2_ (%)	95.6 ± 4.3	94.8 ± 3.9
Minimum SpO_2_ (%)	87.2 ± 6.7	88.9 ± 8.1
AHI (events/h)	8.5 ± 3.9	7.8 ± 4.4
Sleep architecture (%)		
REM sleep	24.8 ± 8.1	22.2 ± 7.3
Light sleep	52.3 ± 4.6	49.6 ± 9.5
Deep sleep	22.9 ± 9.7	28.2 ± 11.6

*BMI* body mass index, *ASA* American Society of Anesthesiologists physical status, *AHI* apnea/hypopnea index, *REM* rapid-eye movement

**Table 2:** The characteristics of operation and anesthesia

	Dexmedetomidine (*n* = 26)	Propofol (*n* = 24)	*P* value
Operation time (min)	147.5 ± 38.9	148.2 ± 43.2	0.952
Anesthesia time (min)	191.0 ± 50.2	201.0 ± 54.2	0.501
Recovery time (min)	44.4 ± 16.9	8.4 ± 7.1	< 0.001
PACU time (min)	37.5 ± 13.1	35.8 ± 11.3	0.627
Phenylephrine (μg)	19.2 ± 8.0	16.3 ± 8.2	0.212
Ephedrine (mg)	1.5 ± 2.4	1.5 ± 3.5	1.000
Atropine (mg)	0.04 ± 0.14	0.02 ± 0.10	0.567

*PACU* post-anesthesia care unit

During the intraoperative sedation period, 3 patients (11.5%; 95% Confidence interval, 2.4–33.7%) and 10 patients (41.7%; 95% Confidence interval, 20.0–76.6%) exhibited signs of upper airway obstruction in the dexmedetomidine and propofol group, respectively (*P* = 0.035). An artificial airway was inserted in 1 patient (3.8%; 95% Confidence interval, 0.1–21.4%) and 5 patients (20.8%; 95% Confidence interval, 6.8–48.6%) in the dexmedetomidine and propofol groups, respectively (*P* = 0.093).

**Fig. 2:**
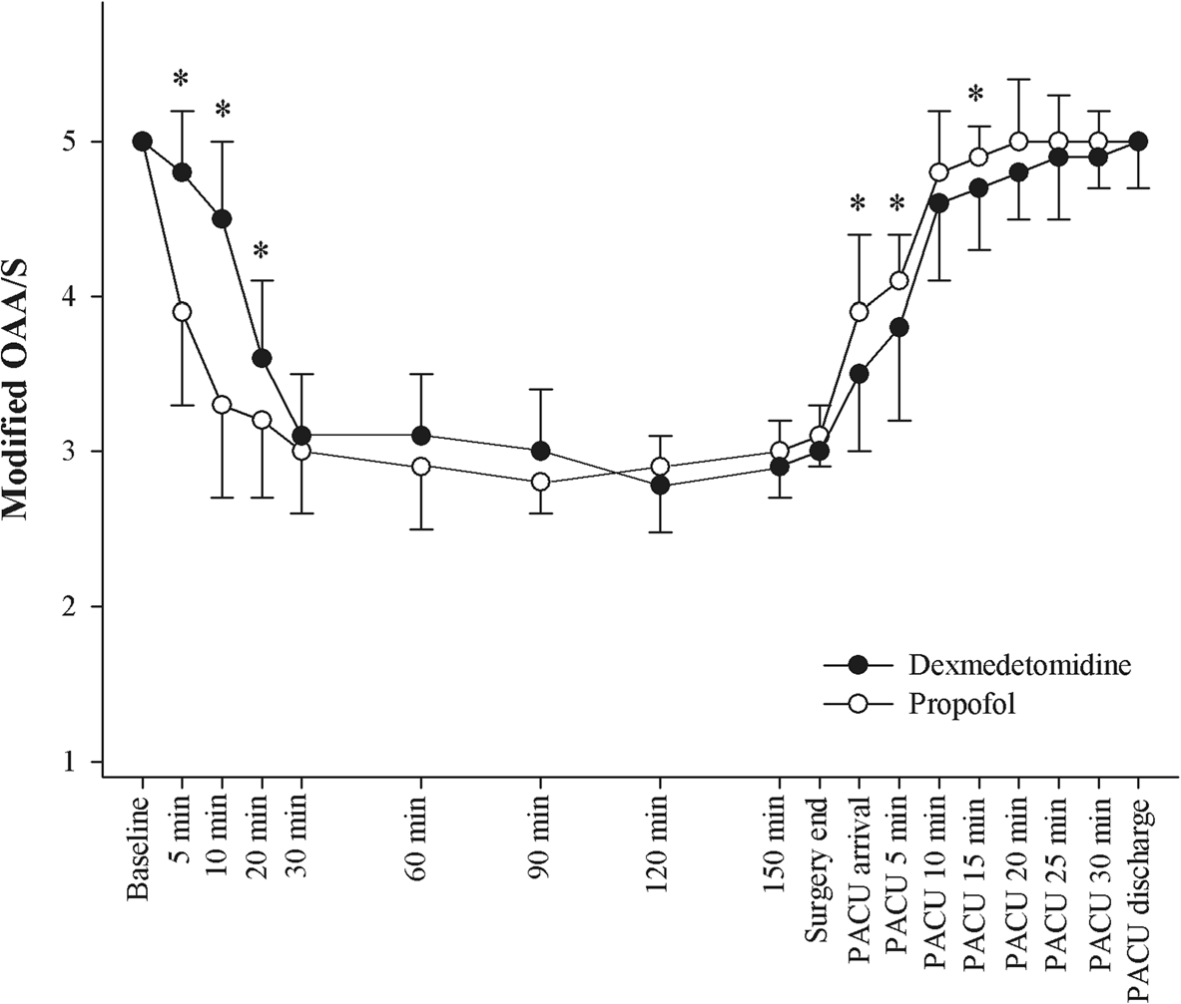
The change of modified Observer’s Assessment of Alertness/Sedation (OAA/S) scale. A stable mid-modified OAA/S scales were presented at 30 min intervals. PACU, post-anesthetic care unit

Figure 2 showed the modified OAA/S scale during sedation and the PACU stay. In the dexmedetomidine group, the modified OAA/S scale was higher during the first 20 min; however, it was maintained at similar levels between the two groups during the remainder of the operation period. The modified OAA/S scale recovered slower in the dexmedetomidine group than in the propofol group in the PACU. The recovery time was significantly longer in the dexmedetomidine group than in the propofol group (44.4 ± 16.9 min in the dexmedetomidine group vs. 8.4 ± 7.1 min in the propofol group, *P* < 0.001). However, the duration of the PACU stay was similar in the two groups (37.5 ± 13.1 min in the dexmedetomidine group vs. 35.8 ± 11.3 min in the propofol group, *P* = 0.627).

**Fig. 3:**
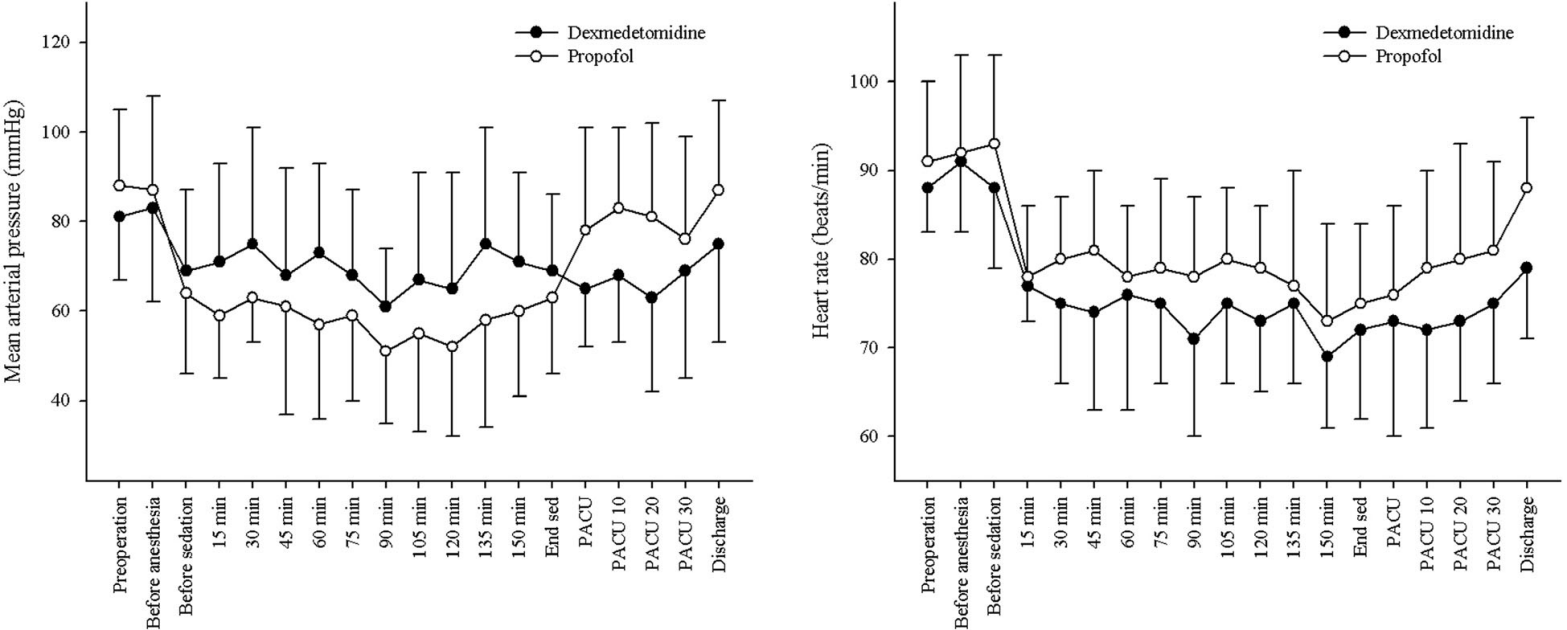
Changed of mean arterial pressure and heart rate during sedation and postoperative period, PACU, post-anesthetic care unit

Changes in mean arterial pressure and heart rate were comparable between the two groups during sedation (Fig. 3). However, intraoperative and postoperative mean arterial pressures demonstrated different patterns. The intraoperative mean arterial pressure was higher in the dexmedetomidine group, while the postoperative mean arterial pressure was higher in the propofol group.

## Discussion

Patients enrolled in the present study had a mild degree of OSA; consequently, they were vulnerable to the occurrence of upper airway obstruction during sedation. We found that there were fewer occurrences of upper airway obstruction during sedation in the dexmedetomidine group than in the propofol group. The incidence of artificial airway insertion was numerically lower in patients who were administered dexmedetomidine, although the difference was not statistically significant.

It has been reported that patients experience at least mid-level anxiety after spinal anesthesia [16]. Thus, sedation during surgery under regional anesthesia is required to reduce patient anxiety and/or stress. However, sedation remains a challenging task for anesthesiologists because there is a possibility of respiratory complications, including upper airway obstruction, respiratory depression, hypercapnia, and hypoxia [17, 18].

In addition to propofol and benzodiazepine, which have both been universally selected for intraoperative sedation, dexmedetomidine can be administered for intraoperative sedation in regional anesthesia [15]. While dexmedetomidine is known to cause less respiratory depression [10], there are limited studies reporting the safety of dexmedetomidine sedation for adult patients with respect to OSA. It was previously reported that dexmedetomidine sedation resulted in less upper airway obstruction in pediatric patients during radiological evaluation compared with propofol sedation, regardless of the presence of OSA [19, 20]. Moreover, it has also been found that there was no dose-dependent change in airway dimensions during dexmedetomidine sedation [21]. In adults, however, there is little evidence that dexmedetomidine is more advantageous for the maintenance of airway patency under sedation. Results of the present study contribute to a better understanding that dexmedetomidine has significantly less effects on upper airway obstruction compared with propofol in adults with mild OSA.

On the other hand, Watt et al. reported that there was no benefit of dexmedetomidine over propofol in deep sedation regarding upper airway obstruction [22]. Patients sedated with propofol also maintained spontaneous respiration; therefore, dexmedetomidine sedation may not be the only attributing factor to decreased occurrence of upper airway obstruction in the dexmedetomidine group.

The exact mechanism of how dexmedetomidine maintains airway patency warrants further investigation. Previously, it was reported that the anteroposterior diameter of the pharynx at the level of the soft palate decreased after propofol administration [23], and pharyngeal dysfunction was also observed [24]. However, Mahmoud et al. also reported that airway dimensions tended to increase slightly in children with OSA by dexmedetomidine, while propofol had a contrary effect [25]. Although their findings failed to reach statistical significance, we are informed that increased airway dimension induced by dexmedetomidine can result in a reduced incidence of upper airway collapse in patients with OSA. Their results are noteworthy, because it has already been established that patients with OSA have a greater risk for upper airway collapse from anesthesia [26].

Although intraoperative sedation level was maintained at modified OAA/S scale 3 in both groups, it took more time for the dexmedetomidine group to recover to a modified OAA/S scale 5, as reported by others [27, 28]. However, we stopped the infusion of dexmedetomidine approximately 30 min before finishing the operation, and this practice helped overcome the extended recovery by dexmedetomidine and did not prolong the PACU stay.

Dexmedetomidine acts as a selective α2 adrenergic receptor agonist and is known to cause hypotension and bradycardia due to decreased centrally mediated sympathetic tone [29]. We did not find any significant differences in the mean arterial pressure and heart rate. However, we found that the mean intraoperative arterial pressure was maintained at a rather higher level in patients sedated with dexmedetomidine. Sympathetic tone was already decreased by spinal anesthesia; therefore, it appears that hypotension was not aggravated by dexmedetomidine. On the other hand, propofol has a direct cardiovascular depressive effect [30]; therefore, patients in the propofol group appeared to exhibit lower mean arterial pressure during the intraoperative sedation period. Due to the short half-life of propofol, the mean arterial pressure increased in the PACU after discontinuing infusion in the propofol group. As expected, dexmedetomidine has a longer half-life than propofol; therefore, mean arterial pressure was maintained at a lower level by continuously blocking central sympathetic outflow in the PACU in the dexmedetomidine group.

The present study had several limitations. The main drawback was the composition of the patients enrolled. We only recruited patients with mild OSA based on Watch-PAT 200 analyses. Thus, it is uncertain whether dexmedetomidine sedation is advantageous to patients with moderate or severe OSA. In addition, patients with a body mass index ≥35 kg/m^2^ were excluded from our study. Morbid obesity is a major risk factor for OSA during sedation; hence, intraoperative sedation should be carefully performed for patient safety. Until now, it is not known which drugs are most suitable for sedation of morbid obese patients. Those with a body mass index ≥35 kg/m^2^ should be included in future studies. Next, the number of patients was insufficient to draw a conclusion with sufficient power regarding the issue of artificial airway insertion. In our study, an artificial airway was used for patients whose airway obstruction was not solved by a positioning aid. Therefore, these patients can be considered to be at higher risk, and their characteristics need to be evaluated separately. Further studies include larger populations will be required. Finally, the investigator assessing the outcomes could not be blinded to the group arrangement because of the characteristics of propofol and dexmedetomidine. However, the primary outcome- upper airway obstruction- was the objective sign presented on the patient’s monitor. Therefore, a bias toward dexmedetomidine or propofol did not appear to be involved in this study.

## Conclusion

Dexmedetomidine sedation was shown to be associated with a reduced incidence of obstructive airway events in patients with mild OSA compared with propofol sedation. We recommended dexmedetomidine as a first-line sedative for adult patients with mild OSA. In future studies, this beneficial effect of dexmedetomidine on airway patency during sedation should be investigated in patients with moderate to severe OSA patient and/or morbidly obese patients.

## Abbreviations

ETCO_2_: End-tidal carbon dioxide
OAA/S: Observer’s assessment of alertness/sedation
OSA: Obstructive sleep apnea
PACU: Post-anesthetic care unit
